# LINC01133 inhibits breast cancer invasion and metastasis by negatively regulating SOX4 expression through EZH2

**DOI:** 10.1111/jcmm.14625

**Published:** 2019-09-26

**Authors:** Zhiwang Song, Xia Zhang, Yun Lin, Youzhen Wei, Shujing Liang, Chunyan Dong

**Affiliations:** ^1^ Department of Oncology, Shanghai East Hospital Tongji University School of Medicine Shanghai China; ^2^ Division of Oncology Research Mayo Clinic Rochester MN USA; ^3^ Research Center for Translational Medicine, Shanghai East Hospital Tongji University School of Medicine Shanghai China

**Keywords:** breast cancer, LINC01133, metastasis, SOX4

## Abstract

Mounting evidence highlights long non‐coding RNAs (lncRNAs) as crucial regulators in multiple types of biological processes and contributing to tumourigenesis. LINC01133, located in chromosome 1q23.2, was a recently identified novel lncRNA with a length of 1154nt. It was involved in the development of colorectal cancer and non‐small cell lung cancer. However, its clinical relevance, biological functions and potential molecular mechanism in breast cancer are still unclear. In this study, we found that the LINC01133 expression was significantly down‐regulated in breast cancer samples and was associated with progression and poor prognosis of breast cancer. Further experiments demonstrated that overexpression of LINC01133 inhibited invasion and metastasis in breast cancer both in vitro and in vivo. Mechanistic investigations revealed that LINC01133 repressed SOX4 expression by recruiting EZH2 to SOX4 promoter. Moreover, rescue experiments further confirmed that LINC01133 functional acted as an anti‐oncogene, at least partly, via repressing SOX4 in breast cancer. Taken together, these findings imply that LINC01133 could serve as a novel prognostic biomarker and potential therapeutic target for breast cancer.

## 1 INTRODUCTION

Breast cancer, originated from breast tissue, is the most commonly diagnosed malignant tumour and one of the leading causes of cancer‐related death among women worldwide.^1^, ^2^ Although dramatic advancement has been made in the early diagnosis, and complex treatment such as surgical resection, radio‐chemotherapy, endocrine therapy and immunotherapy, the prognosis of breast cancer patients is still poor due to high rate of lethal distant metastasis.^3^ Therefore, it is urgent for us to identify novel diagnostic and therapeutic marker and unravel the potential mechanism behind the progression and metastasis of breast cancer to improve the prognosis.

With the rapid development of microarray technique and the whole‐genome sequencing technology over the past decades, it is gradually wide accepted that only less than 2% of human genome is protein‐coding genes, whereas more than 90% of human genome is transcribed into non‐coding RNA without encoding proteins.^4^, ^5^, ^6^ Long non‐coding RNAs (lncRNAs), a newly discovered subgroup of non‐coding RNAs, are transcripts longer than 200 nucleotides without apparent protein‐coding ability.^7^ LncRNAs were initially considered to be transcriptional noise because of the lack of the ability to code protein. However, increasing evidence has indicated that lncRNAs can act as a powerful transcriptional and post‐transcriptional mediator to participate in a large number of physiological and pathological processes, including cell differentiation, cell proliferation, cell apoptosis, cell autophagy, cell invasion and chromosome inactivation. Therefore, lncRNAs are implicated in the initiation and development of human cancers.^8^, ^9^, ^10^, ^11^, ^12^

LINC01133, located in chromosome 1q23.2, was a recently identified novel lncRNA with a length of 1154nt. It was reported that LINC01133 was involved in colorectal cancer and non‐small cell lung cancer.^13^, ^14^, ^15^ However, its clinical relevance, biological functions and potential mechanism in breast cancer are still unclear. In this study, we found the LINC01133 expression significantly reduced in breast cancer tissues compared with paired adjacent normal breast tissues. The reduced LINC01133 expression was correlated with progression and poor prognosis of breast cancer. Moreover, we revealed that LINC01133 inhibited invasion and metastasis in breast cancer, partly through binding with EZH2 to mediate SOX4 transcriptional inhibition.

## 2 MATERIALS AND METHODS

### 2.1 Cell lines

The human breast cancer cell lines MDA‐MB‐231, SKBR‐3, MDA‐MB‐468, ZR‐75‐1, BT474, MCF‐7 and T47D and normal breast epithelial cell line MCF‐10A were purchased from American Type Culture Collection (ATCC). MCF10A cells were cultured in DMEM/F12 (Sigma) supplemented with 5% horse serum (Thermo Fisher Scientific), 20 ng/mL EGF (BD Biosciences), 0.5 mg/mL hydrocortisone (Sigma‐Aldrich), 100 ng/mL cholera toxin (Sigma‐Aldrich), 10 mg/mL insulin (Gibco) and pen/strep. MDA‐MB‐231 cells were cultured in Leibovitz's L‐15 medium with 10% FBS at 37 1C without CO2. MDA‐MB‐436, ZR‐75‐1 and BT474 cells were cultured in RPMI‐1640 (Sigma) medium supplemented with 10% FBS. MCF‐7 were cultured in MEM (10% FBS, 1% NEAA, 0.01 mg/mL bovine insulin (Sigma‐Aldrich), 50 units/mL penicillin and 50 μg/mL streptomycin sulphate). T47D were cultured in RPMI medium (10% FBS, 1% NEAA, 50 units/mL penicillin and 50 μg/mL streptomycin sulphate).

### 2.2 RNA extraction and qPCR assays

The total RNA was extracted from tissue samples or cultured cell lines with TRIzol reagent (Invitrogen), according to the manufacturer's protocol. Total RNA (1 μg) was reverse transcribed in a final volume of 20 μL under standard conditions for the PrimeScript RT reagent Kit (TaKaRa). qRT‐PCR was performed using SYBR Premix Ex Taq (TaKaRa) to determine LINC01133 and targets expression levels, following the manufacturer's instructions. Glyceraldehydes‐3‐phosphate dehydrogenase (GAPDH) was used as an internal reference gene to normalize RNA expression levels between different samples for an exact comparison of transcription levels, and then, the relative expression was calculated using the 2^−ΔΔCt^ method. qRT‐PCR and data collection were performed on an ABI 7500 system (Applied Biosystems). The sequences of PCR primers are listed in Table S1.

### 2.3 Clinical samples

A total of 74 paired breast cancer and adjacent normal breast tissues were collected from patients who were diagnosed with breast cancer according to histopathological evaluation and had received surgical resection at Shanghai Tongji Hospital. None of the patients received chemotherapy or radiotherapy or other anticancer treatment prior to surgery. Written consents were obtained from all patients included in this study. The present study was approved by the Research Ethics Committee of Shanghai Tongji Hospital, Tongji University School of Medicine and was performed in accordance with Declaration of Helsinki. The clinical and pathological characteristics of the breast cancer patients are summarized in Table 1.

**Table 1:** Expression of LINC01133 in relation to pathologic and clinical variables

Clinicopathological parameters	N^2^	LINC01133 expression		*χ* ^2^	*P*‐value
		High	Low		
All	74	29	45		
Age (y)				0.307	.579
<50	31	11	20		
≥50	43	18	25		
Tumour size				0.726	.394
<2.5 cm	30	10	20		
≥2.5 cm	44	19	25		
Lymph node metastasis				4.175	.041
Absence	35	18	17		
Presence	39	11	28		
TNM stage				4.912	.027
I‐II	63	28	35		
III	11	1	10		
Menopause				0.005	.943
Pre	31	12	19		
Post	43	17	26		
ER status				1.635	.201
Negative	34	16	18		
Positive	40	13	27		
PR status				1.184	.277
Negative	44	15	29		
Positive	30	14	16		
Her‐2 status				0.083	.774
Negative	27	10	17		
Positive	47	19	28		

### 2.4 Cell proliferation assays

CCK‐8 (Cell counting kit‐8, Dojindo Laboratories, Japan) was adopted to perform cell assays. Briefly, 2000 cells were cultured in a 96‐well plate. We added with 10 μL CCK‐8 solution to each well and then continued to incubate for 2 hours. Finally, we evaluated the spectrophotometric absorbance at 450 nm.

### 2.5 Wound‐healing assay

MDA‐MB‐231 or MCF‐7 cells were seeded in a 6‐well plate and grown to full confluence in a complete medium. The monolayer was scratched with a plastic pipette tip, washed twice with PBS to remove detached cells and incubated in serum‐free medium for up to 24 hours. The wounded areas were photographed under a microscope equipped with a camera. The changes in cell migration were measured by comparing the difference in wound‐healing assays using EVOS XL Core Imaging System (Invitrogen, Life Technologies). The experiments were repeated at least three times independently.

### 2.6 Transwell assay

Cell invasion assays were performed using the 24‐well BD invasion chambers (BD Biosciences, UK) according to the manufacturer's protocol. Briefly, 5 × 10^4^ cells in 200 μL serum‐free DMEM were seeded per well in the top chambers. 600 μL complete medium was placed to the bottom wells. After culturing for 24 hours, cells in the top chamber were removed with a cotton swab, but the invaded cells were fixed with methanol and stained with 0.1% crystal violet. Finally, five randomly selected fields were counted by using an inverted microscope.

### 2.7 Western blot analysis and antibodies

Proteins were separated by 10% SDS‐polyacrylamide gel electrophoresis, then transferred to Hybond membranes. We blocked membrances for 1.5 hours at room temperature using 5% fat‐free. Then, primary antibodies purchased from Abcam, including SOX4 (ab80261, 1:1000) and β‐actin (ab8227, 1:5000) were incubated overnight at 4°C. And then, the membrane was washed three times with TBST and the secondary antibodies were added for 1 hour at room temperature. Finally, protein was visualized using an enhanced chemiluminescence system and visualized after X‐ray film exposure.

### 2.8 Subcellular fractionation

The PARIS Kit (Life Technologies) was used to conduct the separation of the cytosolic and nuclear fractions in accordance with the manufacturer's protocol.

### 2.9 Fluorescence in situ hybridization (FISH)

RNA FISH was performed according to the manufacturer's instructions. Briefly, MDA‐MB‐231 cells were fixed in 4% formaldehyde for 15 minutes at room temperature. Following, the fixed cells were treated with pepsin and dehydrated through ethanol after washed with PBS and were incubated further with 40 nmol/L of the FISH probe in hybridization buffer. Then, the slide was washed, dehydrated and mounted with Prolong Gold Antifade Reagent with DAPI for detection. Finally, the slides were visualized for immunofluorescence with an Olympus microscope.

### 2.10 RIP

The Magna RIP RNA‐Binding Protein Immunoprecipitation Kit (Millipore) was used to conduct RNA immunoprecipitation (RIP) assays in accordance with the manufacturer's protocol. The EZH2 antibody for this experiment was purchased from Abcam.

### 2.11 RNA‐protein Pull‐down assay, Mass spectrometry

We performed this assay adopting Magnetic RNA‐Protein Pull‐Down Kit (Pierce) according to the manual. Firstly, TranscriptAid T7 High Yield Transcription Kit (ThermoFisher Scientific) was used to yield full length of LINC01133 and labelled using Pierce RNA3′ End Desthiobiotinylation Kit (ThermoFisher Scientific). And then, RNA‐bound beads were added to the cell protein lysate for immunoprecipitation and the beads were washed five times. Finally, the proteins were identified by mass spectrometry (MS) and confirmed by western blotting.

### 2.12 ChIP assays

The EZ‐CHIP KIT (Millipore) was used to conduct the Chromatin immunoprecipitation (ChIP) assays in accordance with the manufacturer's protocol. Firstly, cross‐linked chromatin was broken to 200‐1000 bp fragments using enzymatic digestion. Then, we immunoprecipitate the chromatin adopting anti‐EZH2, anti‐H3K27me3 and anti‐RNA Pol II antibodies. Besides, the normal mouse immunoglobulin G (IgG) was adopted as negative control. Finally, qRT‐PCR was performed.

### 2.13 Mouse tail‐vein assay

To assess the effect of sh‐LINC01133 on breast cancer metastasis, 1 × 10^6^ MDA‐MB‐231 cells infected with sh‐LINC01133 or sh‐NC were injected into the tail vein of female nude mice (ten in each group) to establish a lung metastatic model of breast cancer. Four weeks later, the mice were sacrificed and lung metastasis was quantified by counting the number of tumour foci. The micrometastases in the lung were fixed in formalin and sectioned for H&E staining.

### 2.14 Immunohistochemistry

The tissues were sectioned and subsequently deparaffinized, rehydrated and antigen retrieval. Following blocking with goat serum, SOX4 antibody (1:200; Abcam) was incubated overnight at 4°C and then the tissues were incubated with biotin‐labelled secondary antibodies. The sections were stained and visualized by using 3,3′diaminobenzidine (Maixin Biotech) solution. Images were taken with microscope analysis. All sections were scored blindly by at least two pathologists, and scores were obtained.

### 2.15 Statistical analysis

All statistical analysis was performed with SPSS 21.0 (SPSS). All the data were shown in terms of mean ± SD (standard deviation). The correlation of LINC01133 expression with clinicopathological features was examined by Fisher's Exact Test. Comparisons between two groups were conducted using the Student's *t *test, while ANOVA was performed to evaluate the difference among multi‐groups. The correlation between LINC01133 and SOX4 was analysed using the Pearson's chi‐squared statistics. All *P*‐values were two‐tailed, and *P* < .05 was accepted as statistical significance.

## 3 RESULTS

### 3.1 LINC01133 expression is down‐regulated in human breast tissues and correlated with lymph node metastasis and advanced TNM stage

**Figure 1:**
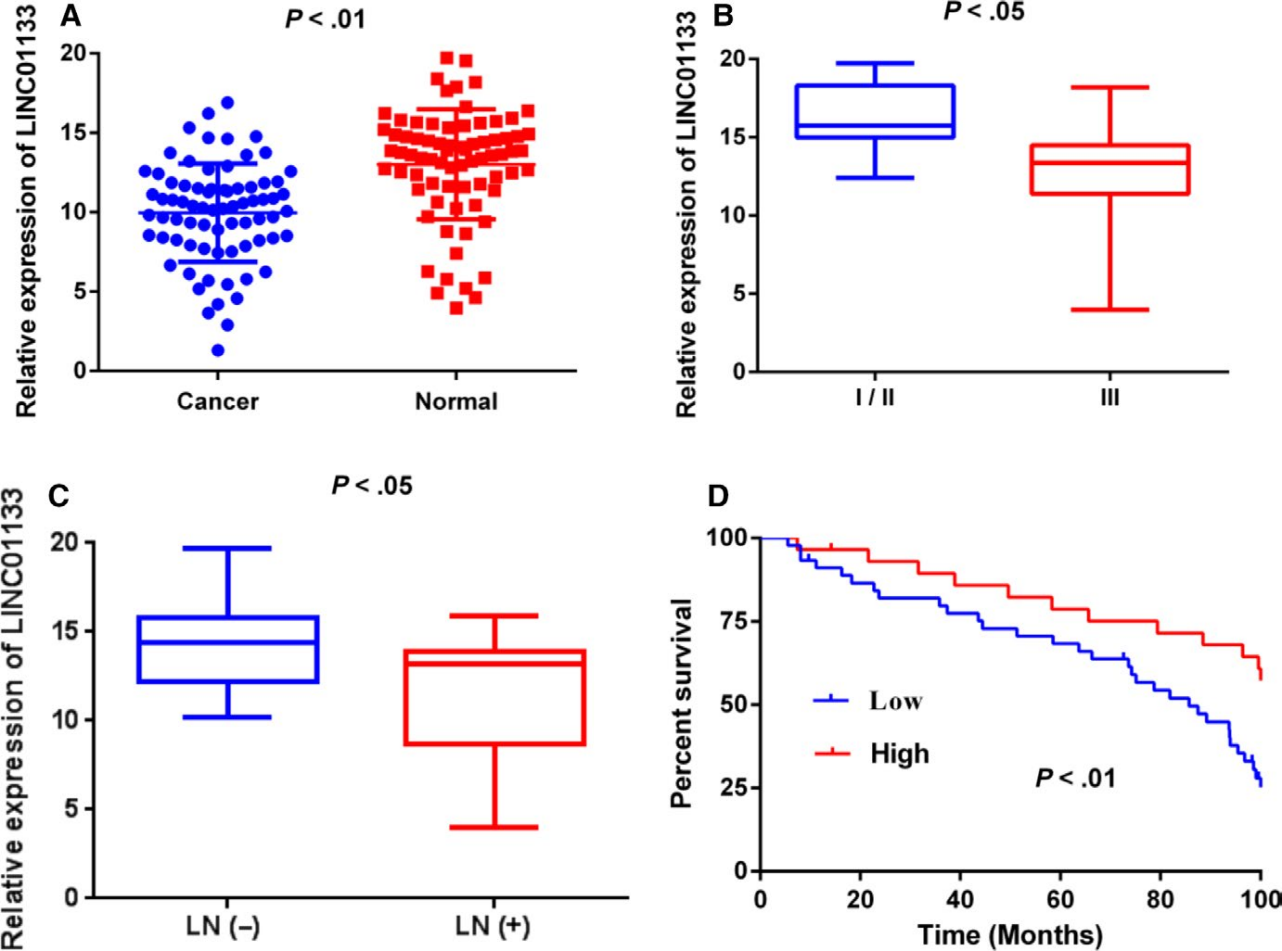
Expression of LINC01133 in breast cancer tissues and its clinical significance. A, LINC01133 expression levels in breast cancer tissues (n = 74) compared with corresponding non‐tumour tissues (n = 74) were examined by qRT‐PCR and normalized against GAPDH expression. B, C, Decreased expression of LINC01133 was significantly correlated with lymph node metastasis and advanced TNM stage. D, Patients with low levels of LINC01133 expression showed reduced survival times compared with patients with HIGH levels of LINC01133 expression

The expression of LINC01133 was examined in 74 human breast cancer tissues and adjacent normal tissues using qRT‐PCR with normalization to GAPDH. The results showed that the expression of LINC01133 was significantly down‐regulated in breast cancer tissues than that in the corresponding non‐cancerous tissues (Figure 1A). Moreover, we evaluated the association of LINC01133 expression with clinicopathological features in breast cancer patients. Our results indicated that the decreased LINC01133 expression was significantly correlated with lymph node metastasis and advanced TNM stage (Figure 1B,C). However, there is no remarkable correlation between LINC01133 expression and other clinical parameters, including patient's age, tumour size, menopause, ER status, PR status and Her‐2 status (Table 1).

### 3.2 Downregulation of LINC01133 predicts a poor prognosis and could be considered as an independent biological predictor for overall survival of breast cancer patients

To investigate the value of LINC01133 expression in the prognosis of breast cancer patients, Kaplan–Meier survival analysis and log‐rank tests were adopted. The results demonstrated that the downregulation of LINC01133 predicted a poor prognosis in breast cancer patients (Figure 1D). Simultaneously, univariate and multivariate Cox regression analyses further revealed that downregulation of LINC01133, together with lymph node metastasis and TNM stage can be regarded as an independent prognostic indicator for breast cancer patients (Table 2).

**Table 2:** Analysis of independent correlation factors of breast cancer prognosis with Cox multivariate regression analysis

Variable	Univariate	Multivariate		
		Hazard	95% CI	*P*‐value
LINC01133 Expression	0.001	2.416	1.071‐3.112	.018
Age	0.147	0.513	0.513‐2.414	.366
Tumour size	0.007	0.684	0.318‐1.409	.217
Lymph node Metastasis	0.003	4.816	1.121‐14.392	.041
TNM stage	0.004	4.133	1.092‐12.885	.032
Menopause	0.019	1.214	0.317‐3.966	.86
ER status	0.016	0.711	0.336‐2.748	.43
PR status	0.075	1.042	0.415‐3.904	.72
Her‐2 status	0.083	0.695	0.312‐2.981	.33

### 3.3 LINC01133 inhibited migration and invasion of breast cancer cells

**Figure 2:**
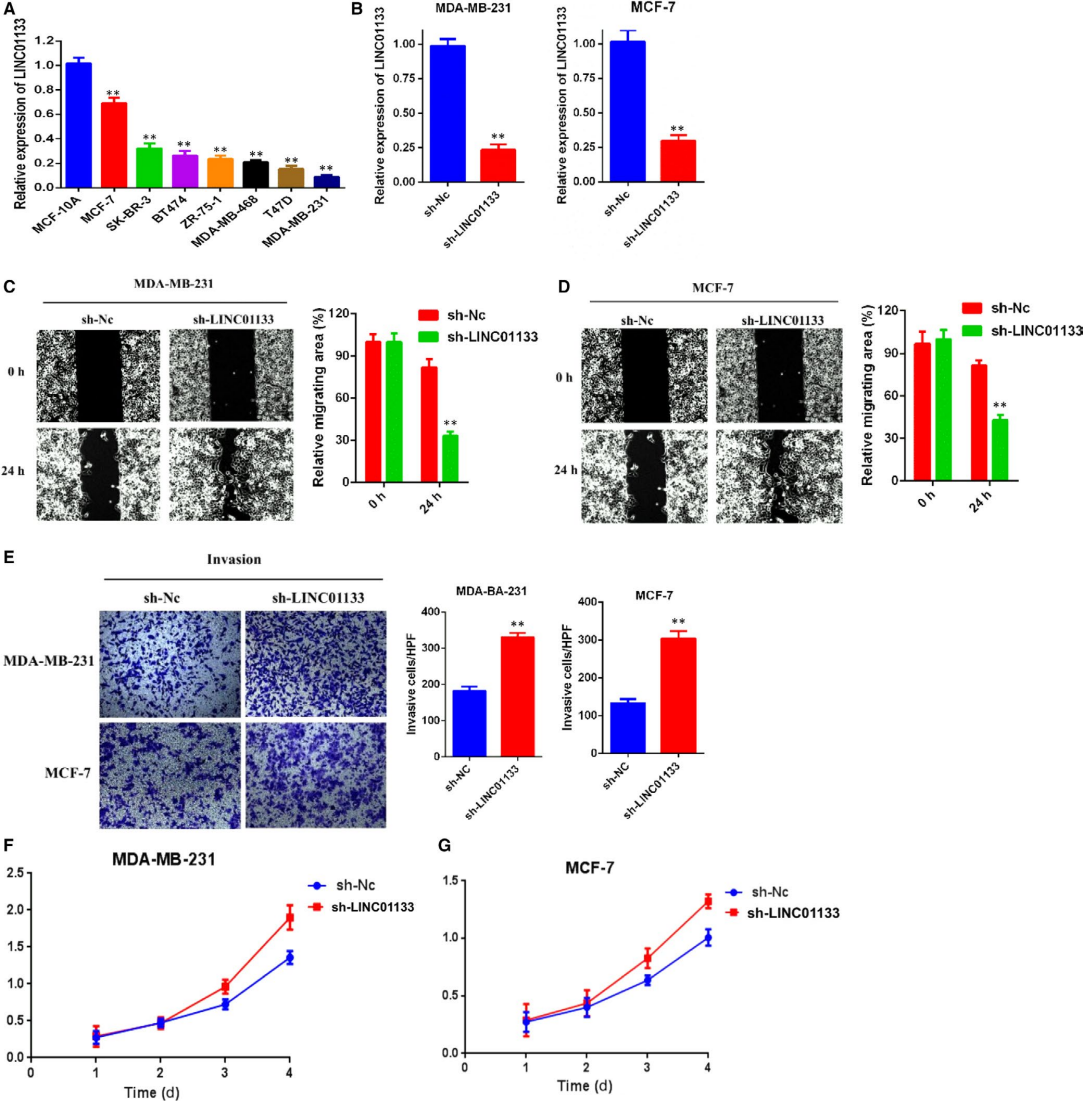
LINC01133 knockdown promotes breast cancer cells migration, invasion and viability in vitro. A, qRT‐PCR analysis of LINC01133 expression in normal breast epithelial cell line (MCF‐10A) and human breast cancer cells. B, Relative expression of LINC01133 in MDA‐MB‐231 and MCF‐7 cells after transfecting with LINC01133 shRNA compared with negative control (Nc). C, D, Wound‐healing assays were performed to assess cell migration following LINC01133 knockdown or Nc in MDA‐MB‐231 and MCF‐7 cells. E, Transwell assays were performed to evaluate cell invasion following LINCO1133 knockdown or Nc in MDA‐MB‐231 and MCF‐7 cells. F, G, CCK‐8 assay of LINC01133 knockdown (shRNAs) and NC breast cancer cells at indicated times

qRT‐PCR analysis was conducted to determine the expression of LINC01133 in 7 human breast cancer cell lines and in the normal breast epithelial cell line MCF‐10A. The results showed that the LINC01133 expression was significantly decreased in breast cancer cell lines compared with that in the normal breast epithelial cell line MCF‐10A (Figure 2A).

**Figure 3:**
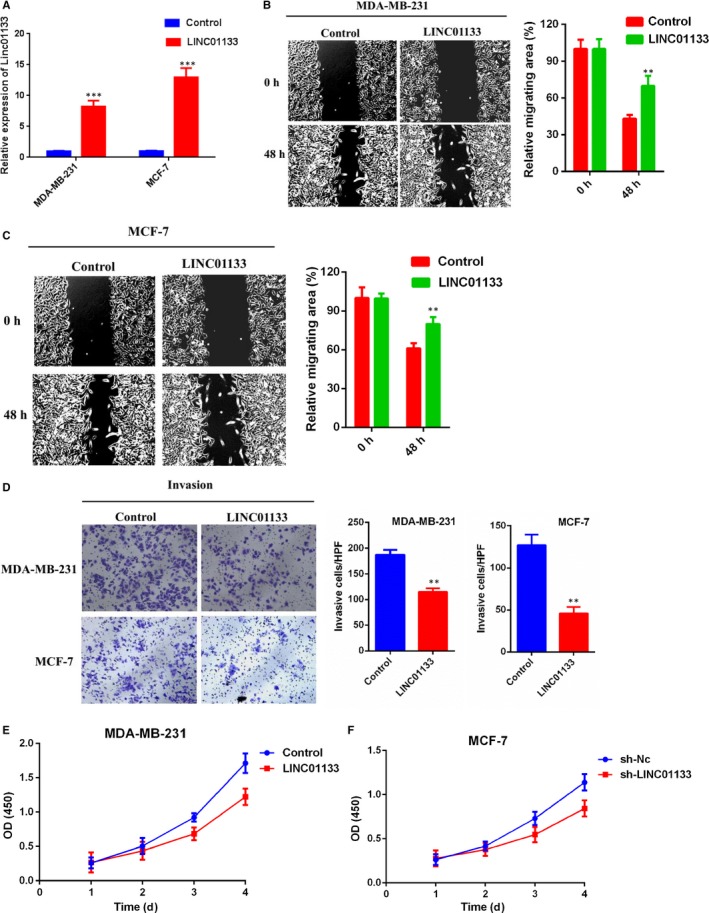
LINC01133 overexpression suppresses breast cancer cells migration, invasion and viability in vitro. A, Relative expression of LINC01133 in MDA‐MB‐231 and MCF‐7 cells transfecting with vector for LINC01133 overexpression compared with vector Nc. B, C, Wound‐healing assays were performed to assess cell migration following LINC01133 overexpression or Nc in MDA‐MB‐231 and MCF‐7 cells. D, Transwell assays were performed to evaluate cell invasion following LINCO1133 overexpression or NC in MDA‐MB‐231 and MCF‐7 cells. E, F, CCK‐8 assay of LINC01133 overexpression and NC breast cancer cells at indicated times

We further explore the functional role of LINC01133 in breast cancer cells. The expression of LINC01133 was significantly decreased after transferred the specific LINC01133 shRNA compared with negative control group (Figure 2B). LINC01133 knockdown markedly promoted the migration (Figure 2C,D) and the invasion (Figure 2E) ability of MDA‐MB‐231 and MCF‐7 cells. In addition, knockdown LINC01133 significantly promoted cell viability of MDA‐MB‐231 and MCF‐7 cells (Figure 2F,G). Conversely, the expression of LINC01133 was remarkably elevated in MDA‐MB‐231 and MCF‐7 cells with stable overexpression of LINC01133 compared negative control (Figure 3A). The overexpression of LINC01133 significantly impaired the migration (Figure 3B,C) and the invasion (Figure 3D) and also inhibited cell viability of MDA‐MB‐231 and MCF‐7 cells (Figure 3E,F). These data revealed that LINC01133 can negatively regulate migration, invasion and viability of breast cancer cells.

### 3.4 LINC01133 suppresses the metastasis of breast cancer cells in vivo

**Figure 4:**
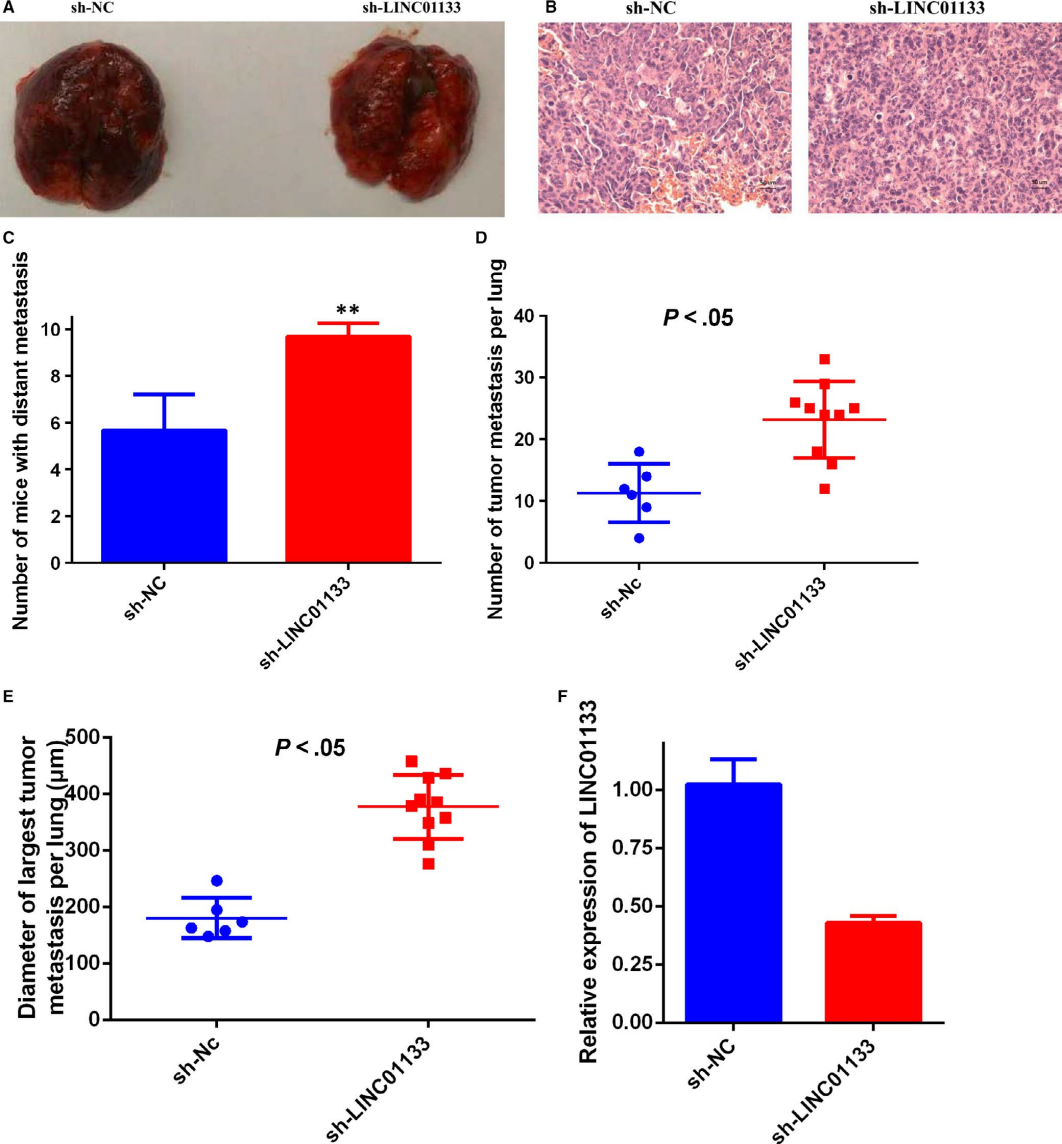
Knockdown of LINC01133 promotes the metastasis of breast cancer in vivo. A, A total 20 BALB/c nude mice aged 4 wk were divided into two groups (10 mice per group), and each mouse was injected with 1 × 10^6^ MDA‐MB‐231 cells infected with sh‐LINC01133 or sh‐Nc into the tail vein. After 4 wk, the mice were killed, and their lungs were removed for assessment of the metastasized tumour nodules. B, H&E staining of the micrometastases in the lung. C, The number of mice with distance metastasis was calculated and compared. D, The numbers of metastatic tumour in nude mice lungs were calculated and compared. E, The diameter of the largest metastatic tumour in nude mice lungs was calculated and compared

In order to investigate the role of LINC01133 on breast cancer metastasis in vivo, MDA‐MB‐231 cells transferred sh‐LINC01133 or sh‐Nc were injected into the tail vein to establish a lung metastatic mice model (Figure 4A,B). Compared with the control group, higher incidence of and more and large pulmonary metastatic lesions were detected in the sh‐LINC01133‐MDA‐MB‐231 group (Figure 4C‐E).

### 3.5 LINC01133 inhibits breast cancer cell metastasis by downregulating SOX4 expression

**Figure 5:**
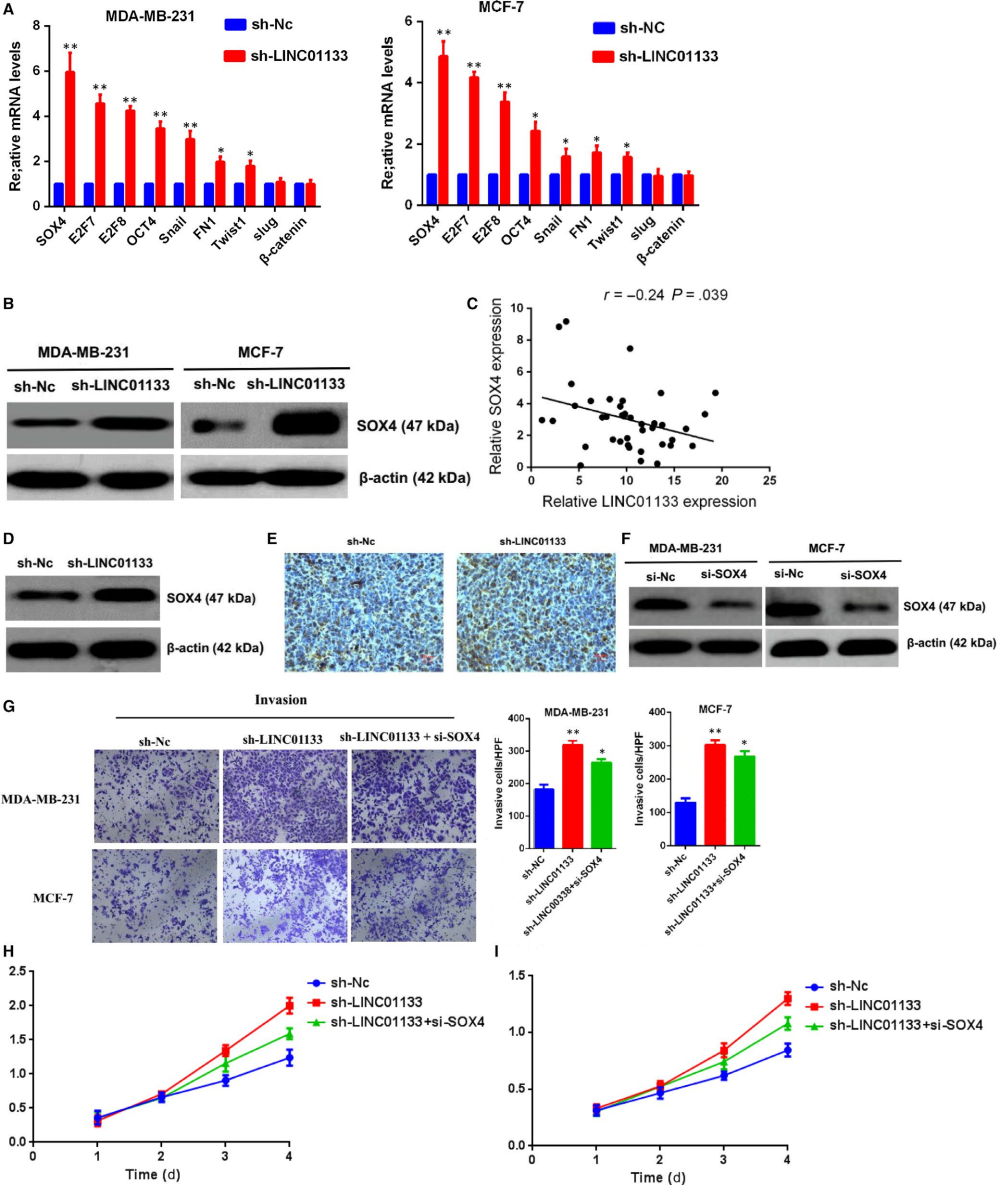
LINC01133 suppresses breast cancer invasion and metastasis by negatively regulating SOX4 expression. A, Expression of the core transcript factors and cell adhesion molecules (SOX4, E2F7, E2F8, OCT4, Snail, FN1, Twist1, slug and β‐catenin) detected by qRT‐PCT in MDA‐MB‐231 and MCF‐7 cells after transfecting with LINC01133 shRNA compared with negative control (Nc). B, Western blot analysis of SOX4 upon LINC01133 knockdown. β‐actin was used as an internal control. C, Correlation of LINC01133 and SOX4 at RNA levels in 40 breast cancer tumour samples. *r* = −.24, *P* = .039 by Pearson correlation analysis. D, Western blot analysis of SOX4 upon LINC01133 knockdown in lung metastatic tumour of nude mice. β‐actin was used as an internal control. E, SOX4 was analysed in lung metastatic tumour of nude mice by immunohistochemical staining. F, Western blot analysis of SOX4 expression in MDA‐MB‐231 and MCF‐7 cells transfecting with si‐SOX4 compared with negative control (Nc). G, Transwell assays in LINC01133 knockdown or NC or sh‐LINC01133 + siSOX4 in MDA‐MB‐231 and MCF‐7 cells. H, I, CCK‐8 assay in LINC01133 knockdown or NC or sh‐LINC01133 + siSOX4 in MDA‐MB‐231 and MCF‐7 cells

To identify the potential downstream targets of LINC01133, qRT‐PCR was performed to examine the expression of the core transcript factors and cell adhesion molecules that have been confirmed to be essential in the migration and invasion of cancer cell (including SOX4, E2F7, E2F8, OCT4, Snail, FN1, Twist1, slug and β‐catenin). Among these analysed genes, SOX4 mRNA level was found to be the most significantly altered after LINC01133 knockdown (Figure 5A). Moreover, western blot analysis further showed that the protein level of SOX4 was markedly increased in response to LINC01133 knockdown MDA‐MB‐231 and MCF‐7 cells (Figure 5B).

Next, we examined the RNA level of SOX4 and LINC01133 in 40 clinical breast cancer samples. The data of qRT‐PCR revealed that the expression of LINC01133 was negatively associated with SOX4 (Figure 5C). Additionally, the expression of SOX4 was increased in LINC01133 knockdown lung metastatic tumours (Figure 5D,E), while the expression of LINC01133 was decreased in LINC01133 knockdown lung metastatic tumours (Figure 5F). These data indicated that the expression of SOX4 was negatively regulated by LINC01133 in breast cancer cells.

It is well‐acknowledged fact that SOX4 contributes to the invasion and metastasis in various types of human cancer, including breast cancer.^16^, ^17^, ^18^ Based on these observations, rescue assays were performed to validate whether SOX4 was involved in the anti‐metastatic function of LINC01133.

After transfection with sh‐LINC01133, MDA‐MB‐231 and MCF‐7 cells were cotransfected with si‐SOX4 (Figure 5F). Our results showed that SOX4 knockdown compromised, at least partially, the effects of LINC01133 on breast cancer invasion (Figure 5G) and cell viability (Figure 5H,I). Taken together, our results indicated that LINC01133 inhibits breast cancer progression at least in part through suppressing SOX4.

### 3.6 LINC01133 suppresses SOX4 expression by recruiting polycomb repressive complex 2 to SOX4 promoter

**Figure 6:**
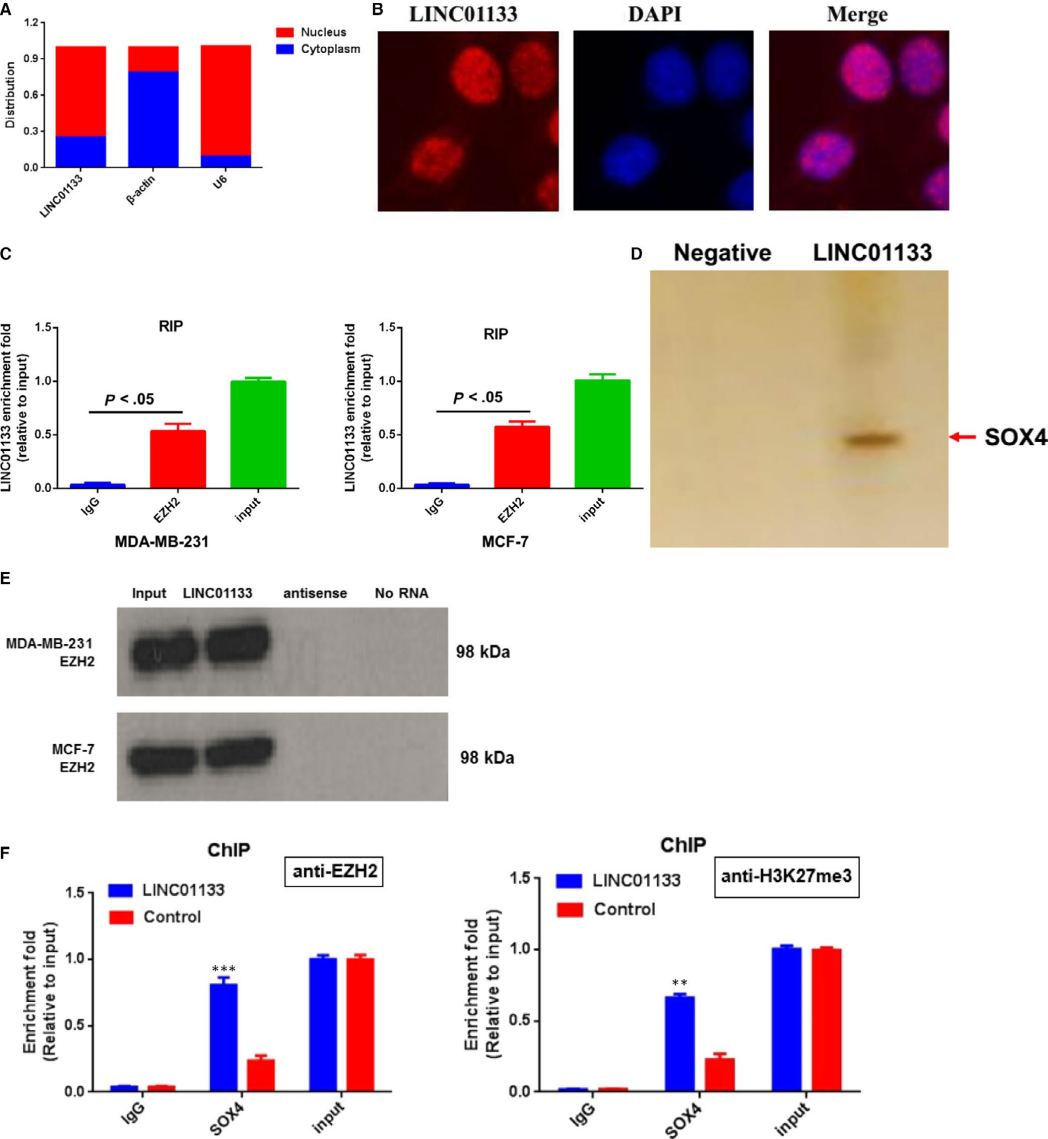
LINC01133 suppresses SOX4 expression by recruiting PRC2 to SOX4 promotor. A, qRT‐PCR analysis of LINC01133 in subcellular fraction of MDA‐MB‐231 cells. U6‐ and β‐actin acted as nucleus and cytoplasm marker, respectively. B, Fluorescence in situ hybridization analysis of the subcellular location of LINC01133 in MDA‐MB‐231 cells. C, RIP assay analysis of the enrichment of LINC01133 to EZH2 in MDA‐MB‐231 and MCF‐7 cells. D, The negative control and LINC01133 transcripts were labelled with biotin and incubated with MDA0MB‐231 lysates, followed by silver staining and mass spectrometry. E, Biotinylated LINC01133 or antisense RNA were incubated with nuclear extracts of MDA‐MB‐231 and MCF‐7 cells, targeted with streptavidin beads, and washed. Then the associated proteins were resolved in a gel. Western blot analysis of the specific association of EZH2 and LINC01133. F, ChIP assays were conducted on SOX4 promoter regions using the indicated antibodies. Enrichment was determined relative to input controls

Firstly, we first determined the subcellular localization of LINC01133 to explore the potential mechanisms by which LINC011331 mediate SOX4 expression. The data of both subcellular fractionation (Figure 6A) and FISH (Figure 6B) revealed that LINC01133 is predominately localized in nucleus. Recently, several studies have supported the novel mechanism that lncRNAs localized in nucleus can recruit polycomb‐group proteins to regulate downstream target genes expression.^19^, ^20^ About 20% of lncRNAs have been reported to associate with Polycomb Repressive Complex 2 (PRC2) and inhibit gene transcription by inducing trimethylation of H3K27.^21^ Therefore, we hypothesized that LINC01133 can suppress the expression of SOX4 in this manner. In order to test this hypothesis, the antibody against EZH2 which was widely acknowledged as an important subunit of the PRC2 complex was adopted to perform RIP assay. Compared with IgG control, a significant enrichment of LINC01133 with EZH2 was found (Figure 6C). Furthermore, we performed RNA pull‐down analysis to explore the interaction between EZH2 and LINC01133 (Figure 6D,E). And then, to further confirm whether LINC01133 inhibited SOX4 transcription by recruiting EZH2 to SOX4 promoter region, we designed SOX4 primers to the promoter region and conducted ChIP assays in LINC01133‐overexpression MDA‐MB‐231 cells. The results revealed that LINC01133 increased the EZH2 and H3K27me3 at the promoter region of SOX4 (Figure 6F), implying that LINC01133 binding to EZH2 to inhibit the transcription of SOX4.

## 4 DISCUSSION

Recently, emerging evidence has indicated that lncRNAs play pivotal roles in breast cancer tumourigenesis and progression, such as ANCR, ROR, MEG3 and H19. ANCR, an important cancer suppressor in breast cancer, can modulate the EZH2 stability and regulate the EMT process and invasion and metastasis of breast cancer.^22^ ROR promoted oestrogen‐independent breast cancer cell growth and activation of MAPK/ERK pathway through regulating the ERK‐specific phosphatase DUSP7.^23^ MEG3 inhibited breast cancer cell proliferation and invasion through regulating E‐cadherin expression by sponging to miR‐421.^24^ A feedback loop constituted of H19, let‐7 and LIN28 played an important role in breast cancer stem cells maintenance.^25^ Our previous study also revealed that lncRNA SUMO1P3 promoted proliferation, migration and invasion of breast cancer cells by negatively regulation of miR‐320a.^26^

In the present study, we identified another lncRNA LINC01133 and founded that its expression was significantly reduced in breast cancer tissues compared with those in the corresponding non‐cancerous tissues. Down‐regulated LINC01133 expression was remarkably associated with progression and poor prognosis in breast cancer patients. Moreover, down‐regulated expression of LINC01133 can be regarded as an independent prognostic indicator for breast cancer patients. These data implied that LINC01133 may have a critical role in the breast cancer progression. Previous studies have reported that LINC01133 was down‐regulated in colorectal cancer.^13^, ^14^ However, Zang et al^15^ reported that LINC01133 was upregulated in non‐small cell lung cancer. These findings are probably because lncRNAs exhibit a more remarkably tissue‐specific expression pattern than protein‐coding genes.^27^, ^28^ And, these data also indicated that LINC01133 had such tissue‐specific expression pattern. To further explore the role of LINC01133 in breast cancer, loss of function assays was performed and found that LINC01133 could promote breast cancer cell migration and invasion in vitro and facilitate breast cancer metastasis in vivo. Conversely, the overexpression of LIC01133 can impair breast cancer cell migration and invasion in vitro. These data indicated that LINC01133 may function as anti‐oncogene and might play vital biological role in the development of breast cancer.

To further determine potential targets of LINC01133 involved in breast cancer invasion and metastasis, we investigate the expression of metastatic regulators and found that both mRNA level and protein level of SOX4 was significantly increased in response to LINC01133 knockdown. Furthermore, we confirmed that the oncogenic effect of LINC01133 knockdown on breast cancer cells is at least partly dependent on the negative regulation of SOX4. Here, for the first time, we showed that LINC01133 exerted anti‐oncogenic functions in breast cancer cells by inhibition of SOX4.

LncRNAs can regulate the expression of target gene via multifarious biological mechanisms, including recruiting chromatin‐modifying enzyme to target gene, mediating the target gene transcription either in cis or in trans, functioning as molecular scaffolds for bind relevant components, and regulating target genes as competing endogenous RNAs via binding with miRNA response elements.^29^, ^30^ To further elucidate the molecular mechanism by which LINC01133 regulates SOX4, nucleocytoplasmic separation experiments and FISH were performed. The result demonstrated that LINC01133 is predominately distributed in nucleus, implying that LINC01133 may play transcriptional regulation role.

Furthermore, these results of RNA pull‐down and RIP assays demonstrated that LINC01133 can bind directly with EZH2 to silence SOX4 expression. ChIP analysis revealed that EZH2 can directly bind to SOX4 promoter regions and induce trimethylation of H3K27 in breast cancer cells. These results indicated that LINC01133 inhibited breast cancer invasion and metastasis in a pattern that is dependent on the negative regulation of the expression of SOX4 through binding to EZH2.

In summary, these findings showed for the first time that LINC01133 was down‐regulated in breast cancer cells and tissues, and the downregulation of LINC01133 is associated with progression and poor prognosis of breast cancer patients. The inhibition role of LINC01133 on breast cancer invasion and metastasis may partly through restraining SOX4 transcription by binding with EZH2. LINC01133 may serve as an important potential target for the diagnosis and treatment of breast cancer. However, further studies are still needed to clarify other potential target of LINC01133 and molecular regulation mechanism in breast cancer.

## CONFLICT OF INTEREST

None.

## AUTHOR CONTRIBUTIONS

ZWS and CYD designed the study. ZWS, XZ, YL and SJL collated the data, carried out data analyses and produced the initial draft of the manuscript. YZW and CYD contributed to drafting and polishing the manuscript. All authors have read and approved the final submitted manuscript.
