# Corrigendum

**DOI:** 10.1111/jcmm.15085

**Published:** 2020-04-20

In ‘LINC01133 inhibits breast cancer invasion and metastasis by negatively regulating SOX4 expression through EZH2’, which was published in volume 23 issue 11, October 2019. The original article contains incorrect Figures 2D, 3C and F. The correct version of the figures is presented below.

**Figure 2:**
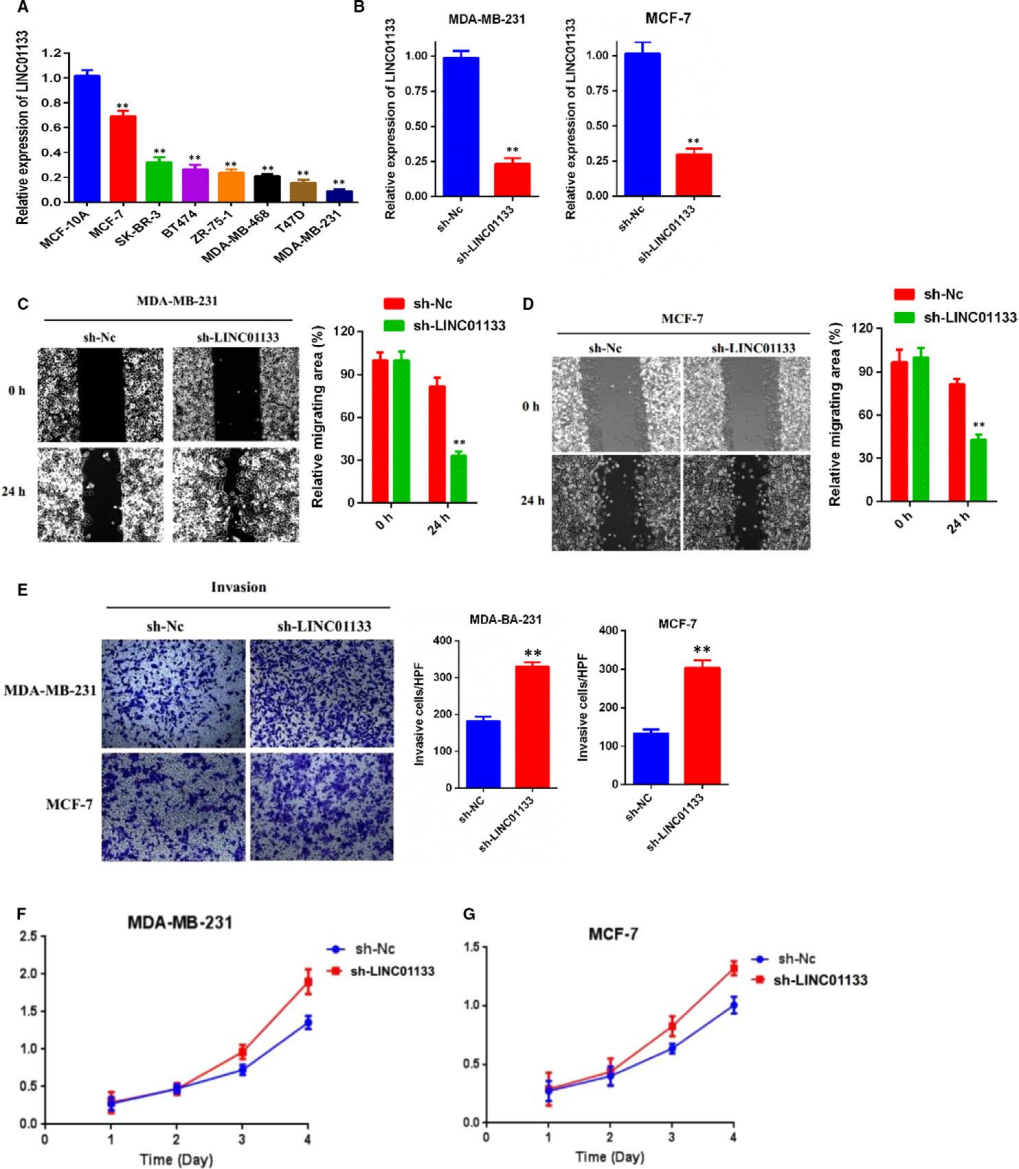
LINC01133 knockdown promotes breast cancer cells migration, invasion and viability in vitro. A, qRT‐PCR analysis of LINC01133 expression in normal breast epithelial cell line (MCF‐10A) and human breast cancer cells. B, Relative expression of LINC01133 in MDA‐MB‐231 and MCF‐7 cells after transfecting with LINC01133 shRNA compared with negative control (Nc). C, D, Wound‐healing assays were performed to assess cell migration following LINC01133 knockdown or Nc in MDA‐MB‐231 and MCF‐7 cells. E, Transwell assays were performed to evaluate cell invasion following LINCO1133 knockdown or Nc in MDA‐MB‐231 and MCF‐7 cells. F, G, CCK‐8 assay of LINC01133 knockdown (shRNAs) and NC breast cancer cells at indicated times

**Figure 3:**
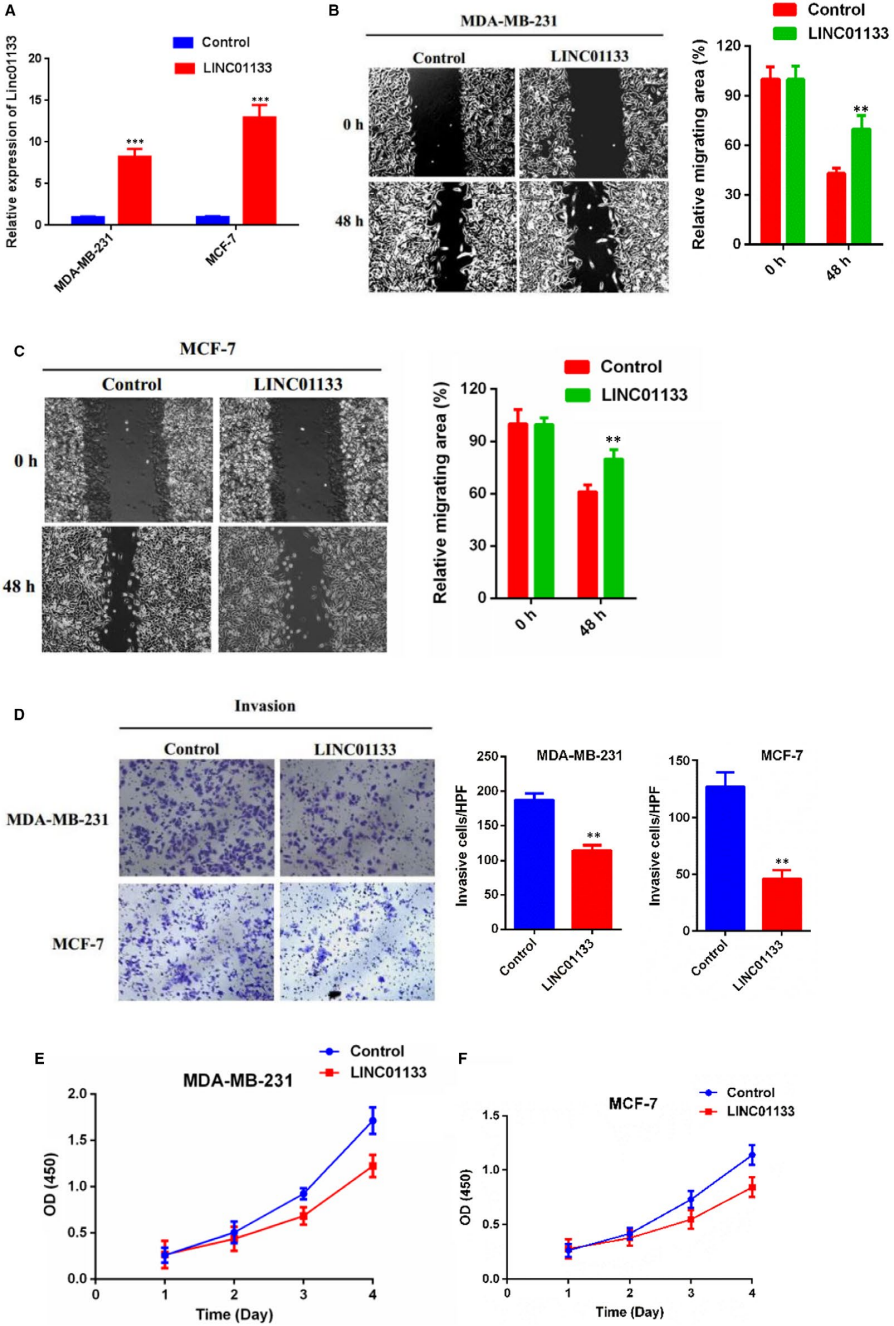
LINC01133 overexpression suppresses breast cancer cells migration, invasion and viability in vitro. A, Relative expression of LINC01133 in MDA‐MB‐231 and MCF‐7 cells transfecting with vector for LINC01133 overexpression compared with vector Nc. B, C, Wound‐healing assays were performed to assess cell migration following LINC01133 overexpression or Nc in MDA‐MB‐231 and MCF‐7 cells. D, Transwell assays were performed to evaluate cell invasion following LINCO1133 overexpression or NC in MDA‐MB‐231 and MCF‐7 cells. E, F, CCK‐8 assay of LINC01133 overexpression and NC breast cancer cells at indicated times
