# Supplementary material for: LINC01133 inhibits breast cancer invasion and metastasis by negatively regulating SOX4 expression through EZH2
Source: J Cell Mol Med. 2019 Sep 26;23(11):7554–65. doi: 10.1111/jcmm.14625 (PMC6815803; doi:10.1111/jcmm.14625)
Supplement: Supplementary file 1 [file JCMM-23-7554-s001.docx]

| Oligos | Sequence |
| --- | --- |
| LINC01133-ChIRP probes | AGCTTTGTTAATGGCTCCTC |
|  | TACTGAGAAACCCAGTTCCT |
|  | CCGGTCATGTTGTTCATCTT |
|  | GGAGAACTTTGCTTCCACTT |
|  | TTTTTGAGGTTCACCTTGCC |
|  | AAACAAACTCAGAGGCACTG |
|  | TTAAGTATTTCTGGGGCCAG |
|  | TAAATGGATCCCTATGCCCT |
| Control-ChIRP probes([1](#_ENREF_1)) | TTAAAGCGAGTGGCAACATG |
|  | TCACGACGTTGTAAAACGAC |
|  | TTAACGCCTCGAATCAGCAA |
|  | TGATCACACTCGGGTGATTA |
|  | TTAATCAGCGACTGATCCAC |
|  | GTTCAGGCAGTTCAATCAAC |
|  | TAAGGTTTTCCCCTGATGCT |
|  | AGATGAAACGCCGAGTTAAC |
| GAPDH-primer-F | ACCACAGTCCATGCCATCAC |
| GAPDH-primer-R | TCCACCACCCTGTTGCTGTA |
| SOX4-primer-F | CAGCGACAAGATCCCTTTCA |
| SOX4-primer-R | GCCGGACTTCACCTTCTTC |
| E2F7-primer-F | GTCGCCAGGTGCCTCTTGTAAG |
| E2F7-primer-R | ATGCTGCCTGTGACACCATGAC |
| E2F8-primer-F | GGAAGCGGTTGGAAGGTGTGAG |
| E2F8-primer-R | AGGAGGCTGAGGCAGGAGAATG |
| OCT4-primer-F | CTGTCGCCTCTCATGCGGATG |
| OCT4-primer-R | CGCGTTCCTCACCACTGTTGG |
| snail-primer-F | CCATCACTGCCAGCCGTTGTC |
| snail-primer-R | GCCGGACTCTTGGTGCTTGTG |
| FN1-primer-F | GGAGGCTGAGGCGGAAGGATC |
| FN1-primer-R | CAGTGTGCCAGGTGCTGAAGC |
| Twist1-primer-F | CCGTCCGTCCTCCTGCTCTC |
| Twist1-primer-R | GTCTGGCTCTTCCTCGCTGTTG |
| LINC01133-primer-F | CCTAATCTCACCACAGCCTGG |
| LINC01133-primer-R | TCAGAGGCACTGATGTTGGG |
| SOX4-siRNA-sense | CCAGAAAUACUUAAUUCAACU |
| SOX4-siRNA-antisense | UUGAAUUAAGUAUUUCUGGGG |
| LINC01133-shRNA-F | CCGGAACCAGAAAUACUUAAUUCAACTCGAGTTGTTGTTTTCTGGTTTTTTTG |
| LINC01133-shRNA-R | AATTCAAAAAAACCAGAAAUACUUAAUUCAACTCGAGTTGTTGTTTTCTGGTT |

**Supplementary materials**

Oligos used in this study

**Materials and Methods**

**Cell lines.** The human breast cancer cell lines MDA-MB-231, SKBR-3, MDA-MB-468, ZR-75-1, BT474, MCF-7, and T47D and normal breast epithelial cell line MCF-10A were purchased from American Type Culture Collection (ATCC, USA). MCF10A cells were cultured in DMEM/F12 (Sigma, St. Louis, MO, USA) supplemented with 5% horse serum (Thermo Fisher Scientific, Waltham, MA USA), 20 ng/ ml EGF (BD Biosciences, Franklin Lakes, NJ, USA), 0.5 mg/ml hydrocortisone (Sigma-Aldrich), 100 ng/ml cholera toxin (Sigma-Aldrich), 10 mg/ml insulin (Gibco) and pen/strep. MDA-MB-231 cells were cultured in Leibovitz’s L-15 medium with 10% FBS at 37 1C without CO2. MDA-MB-436, ZR-75-1, and BT474 cells were cultured in RPMI-1640 (Sigma) medium supplemented with 10% FBS. MCF-7 were cultured in MEM (10% FBS, 1% NEAA, 0.01 mg/ml bovine insulin (Sigma-Aldrich, St. Louis, MO, USA), 50 units/ml penicillin and 50 μg/ml streptomycin sulfate). T47D were cultured in RPMI medium (10% FBS, 1% NEAA, 50 units/ml penicillin and 50 μg/ml streptomycin sulfate).

**RNA extraction and qPCR assays.** The total RNA was extracted from tissue samples or cultured cell lines with TRIzol reagent (Invitrogen, Grand Island, NY, USA), according to the manufacturer’s protocol. Total RNA (1ug) was reverse transcribed in a final volume of 20 μl under standard conditions for the PrimeScript RT reagent Kit (TaKaRa, Dalian, China). qRT-PCR was performed using SYBR Premix Ex Taq (TaKaRa, Dalian, China) to determine LINC01133 and targets expression levels, following the manufacturer’s instructions. Glyceraldehydes-3-phosphate dehydrogenase (GAPDH) was used as an internal reference gene to normalize RNA expression levels between different samples for an exact comparison of transcription levels, and then the relative expression was calculated using the 2^-ΔΔCT^ method. qRT-PCR and data collection were performed on an ABI 7500 system (Applied Biosystems, Foster City, CA, USA). The sequences of PCR primers are listed in Supplementary Table S1.
